# Personalized ophthalmology

**DOI:** 10.1111/cge.12389

**Published:** 2014-02-09

**Authors:** LF Porter, GCM Black

**Affiliations:** a Centre for Genomic Medicine, Institute of Human Development, Faculty of Medical and Human Sciences, University of Manchester, Manchester Academic Health Science Centre (MAHSC), Saint Mary's HospitalManchester, UK; b Manchester Royal Eye Hospital, Department of OphthalmologyManchester, UK; c Centre for Genomic Medicine, Central Manchester University Hospitals NHS Foundation Trust, MAHSC, Saint Mary's HospitalManchester, UK

**Keywords:** gene therapy, genetics, genomics, inherited eye disease, molecular diagnosis, personalized medicine, personalized ophthalmology, next-generation sequencing, targeted therapies

## Abstract

Porter L.F., Black G.C.M. Personalized ophthalmology. Clin Genet 2014: 86: 1–11. © 2014 The Authors. *Clinical Genetics* published by John Wiley & Sons A/S. Published by John Wiley & Sons Ltd., 2014

Ophthalmology has been an early adopter of personalized medicine. Drawing on genomic advances to improve molecular diagnosis, such as next-generation sequencing, and basic and translational research to develop novel therapies, application of genetic technologies in ophthalmology now heralds development of gene replacement therapies for some inherited monogenic eye diseases. It also promises to alter prediction, diagnosis and management of the complex disease age-related macular degeneration. Personalized ophthalmology is underpinned by an understanding of the molecular basis of eye disease. Two important areas of focus are required for adoption of personalized approaches: disease stratification and individualization. Disease stratification relies on phenotypic and genetic assessment leading to molecular diagnosis; individualization encompasses all aspects of patient management from optimized genetic counseling and conventional therapies to trials of novel DNA-based therapies. This review discusses the clinical implications of these twin strategies. Advantages and implications of genetic testing for patients with inherited eye diseases, choice of molecular diagnostic modality, drivers for adoption of personalized ophthalmology, service planning implications, ethical considerations and future challenges are considered. Indeed, whilst many difficulties remain, personalized ophthalmology truly has the potential to revolutionize the specialty.

## Personalized medicine, why now?

The term ‘personalized medicine’ is used in a broad sense to encompass all approaches used to tailor healthcare to the needs of individual patients. The medical profession has always practiced personalized medicine; considering factors such as age, sex, and family history in approaching diagnosis and taking into account co-morbidities, lifestyle, family and socio-economic circumstances 1. However, over the past 60 years medicine has been under increasing pressure to adopt a more scientific approach exemplified by the advent of evidence-based-medicine, a concept that was further developed in the early 1990s, focusing on evidence-based clinical decision-making (1, S1–S2, Appendix S1, Supporting Information). The completion of the human genome project and extraordinary changes brought about by novel ‘next generation sequencing’ (NGS) technologies now heralds an era in which genomics promises to accelerate the personalization of the science of medicine (2 3, S3). A focus on the individual patient is receiving renewed attention (S4–S5). The genomics revolution will exert a profound influence over clinical practice (2 4, 5, S6) and promises to transform ophthalmology.

## Personalizing ophthalmology

**Figure 1:**
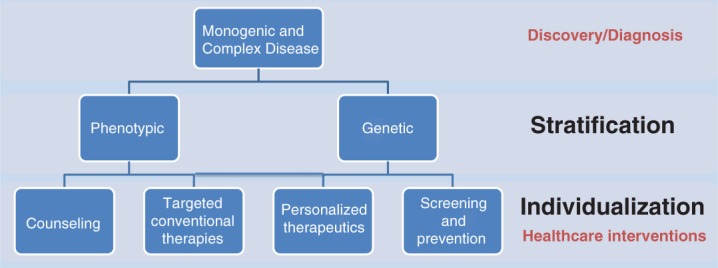
Personalized ophthalmology: from stratified medicine to individualized therapies, including a translational process from gene discovery and molecular diagnostic advances to healthcare interventions.

Ophthalmology has been an early adopter of personalized medicine. Currently, the application of genetic technology is heralding the development of individualized treatments for some inherited monogenic eye diseases (6 7, S7–S12), and promises to alter prediction, diagnosis and management of complex diseases such as age-related macular degeneration (AMD) (8, S13). The development of personalized ophthalmology will necessarily be underpinned by a deep scientific understanding of the molecular basis of eye disease, which forms the foundation for development of tailored approaches to preventative, diagnostic and therapeutic strategies. Two important areas of focus are required for the successful adoption of personalized approaches in the ophthalmic clinic: *stratification* and *individualization* (Fig. 1).

Stratification of disease, achieved at phenotypic and genotypic levels, is already being successfully applied in many areas of ophthalmology, most notably inherited eye disease (IED), accelerated by novel imaging technologies and relying on so called ‘deep phenotypic’ clinical assessment and molecular diagnosis (S14–S16) (Table1). Dissecting the molecular basis of IED stratifies what was often considered a single disease entity into different disease subtypes. Phenotypic stratification in genetically heterogeneous monogenic diseases, such as retinitis pigmentosa (RP) for example, enables identification of gene-specific phenotypes. In complex diseases such as age-related macular degeneration (AMD), disease stratification identifies endophenotypes.

**Table 1:** Advantages of molecular diagnosis in disease stratification

Advantages of broadly available molecular diagnosis for disease stratification
- Provision of precise clinical diagnosis
- Establishment of pattern of inheritance
- Provision of accurate prognosis
- Counseling for facilitated decision making
- Construction of disease registries
- Early access to personalized treatments
- Enhanced access to clinical trials

Enormous progress has been made over the last three decades using genetic technologies as a research platform to dissect the molecular pathways underpinning both monogenic and complex eye disease. The adoption of NGS technologies is accelerating such discoveries, particularly in monogenic disorders, underpinned by exome-based sequencing (3 4, S3). For example, NGS discovery of disease-mutated genes, both novel and known, in families with a history of AR disease (including ocular disease) successfully identifies a mutated disease-causing variant in around 70% of cases in a highly consanguineous Middle Eastern population (4, S17). NGS technologies are revolutionizing access to molecular diagnosis for many patients with IED, with particular benefits for conditions characterized by genetic and phenotypic heterogeneity such as inherited retinal diseases (IRD), congenital cataract (CC) and inherited optic nerve disorders (5, S18–S20). This is by no means restricted to research with NGS now becoming established in a clinical diagnostic setting for a number of ocular phenotypes (5, S21).

**Figure 2:**
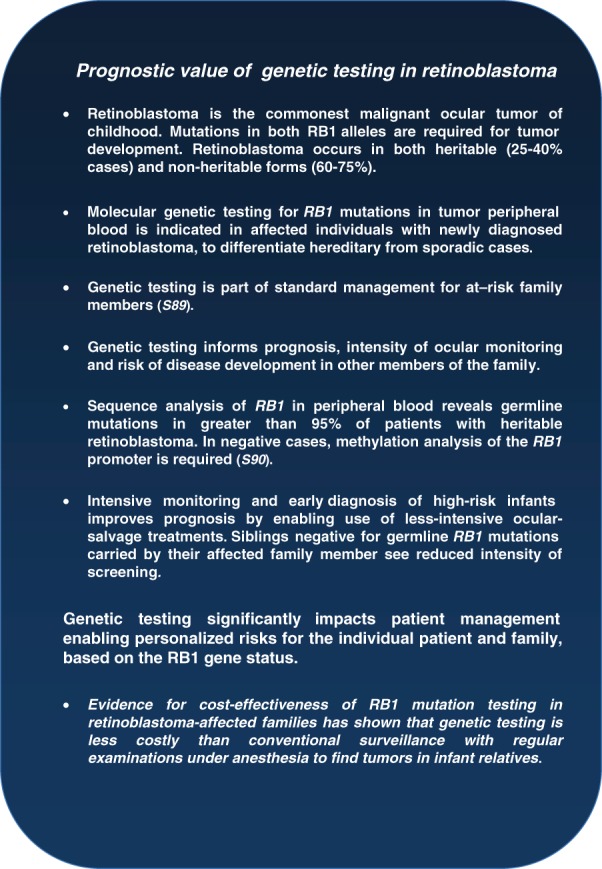
Tailoring of care for retinoblastoma.

With individualization, a personalized approach enables treatment to be based on an individual's genetic or biomarker profile (2 4, 5, S6), ultimately this approach encompasses all aspects of patient management from optimized genetic counseling and conventional therapies, to trials of novel DNA-based therapies. The clinical implications of disease stratification and individualization are discussed in the following sections. These twin strategies promise to drive renewed focus on preventative healthcare, based on a biomedically-driven approach to predictive and pre-symptomatic risk assessment (4, S6, S22–S23) that will be applied to both monogenic and complex ophthalmic diseases alike (Fig. 2).

## Stratification

### Monogenic ocular disorders – NGS for diagnosis of genetically heterogeneous conditions – clinical implications

#### Inherited retinal diseases (IRDs)

**Figure 3:**
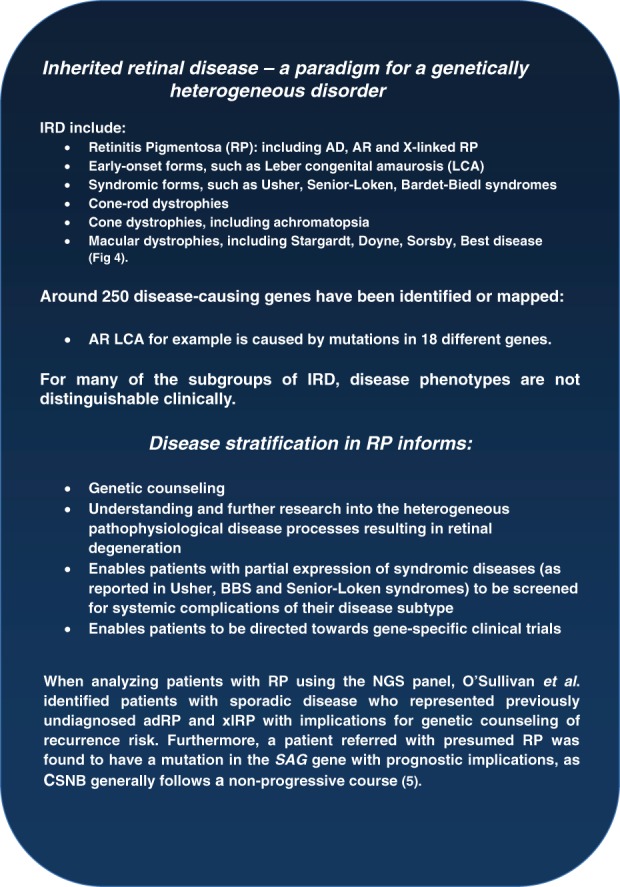
Genetic and phenotypic heterogeneity in inherited retinal diseases (IRD): an example of disease stratification. Molecular diagnosis alters disease management.

**Figure 4:**
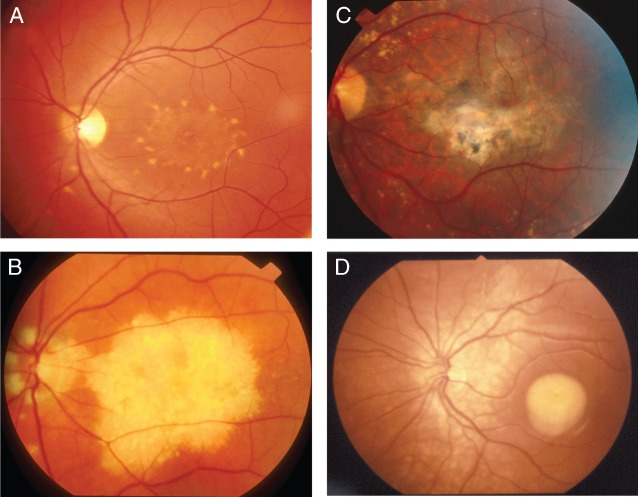
Monogenic macular dystrophies. A heterogenous group of disorders, each caused by mutations in a single gene. Incomplete penetrance and variability in disease expression complicate diagnosis. (**a**) Stargardt disease (STGD1) is the most prevalent juvenile retinal dystrophy. With an autosomal recessive (AR) pattern of inheritance, STGD1 is associated with rapid central vision loss. Mutations in *ABCA4* give rise to STGD1. Defective ABCA4 protein leads to the accumulation of A2E lipofuscin in retinal pigment epithelium (RPE) cells, which is toxic in high concentrations. The accumulation of lipofuscin can be seen in and around the macula as yellowish white flecks (S91). (**b**) Doyne honeycomb retinal degeneration (Malattia Leventinese) is a dominant macular dystrophy caused by mutations in *EFEMP1*.Affected individuals present with drusen in the macula and around the edge of the optic nerve head (S92). (**c**) Sorsby fundus dystrophy results from mutations in *TIMP3* and resembles neovascular age-related macular degeneration (AMD) with an age of onset in early adulthood (S93). (**d**) Best vitelliform macular dystrophy (BVMD) is one of the ‘bestrophinopathies’ caused by AD mutations in *BEST1*, encoding bestrophin-1, a calcium-activated chloride channel. Accumulation of subretinal fluid and vitelliform material originating from the outer photoreceptors is thought to cause RPE overload, leading to photoreceptor and RPE dysfunction (S94).

IRDs are a genetically and phenotypically heterogeneous group of conditions characterized by rod and cone photoreceptor degeneration (S24). Patients with IRD pose a compelling case for personalized ophthalmology and show the need for widespread genetic testing (9, S25–S26), particularly because of the advent of gene-based clinical trials 7. The nature of phenotypic and genetic heterogeneity amongst IRD is shown in Figs 3 and 4. Because conventional sequencing strategies are impractical in such contexts, NGS-based protocols are required to identify disease-causing mutations. In the clinical setting this is being achieved using custom-based target enrichment panels or whole exome sequencing approaches with a targeted bioinformatics analysis focusing upon genes causing IRD (5, S18). O'Sullivan et al. for example analyzed 105 genes in 50 patients with IRD, successfully identifying a disease-causing mutations in 50–55% of patients 5.

#### Early onset glaucoma

**Figure 5:**
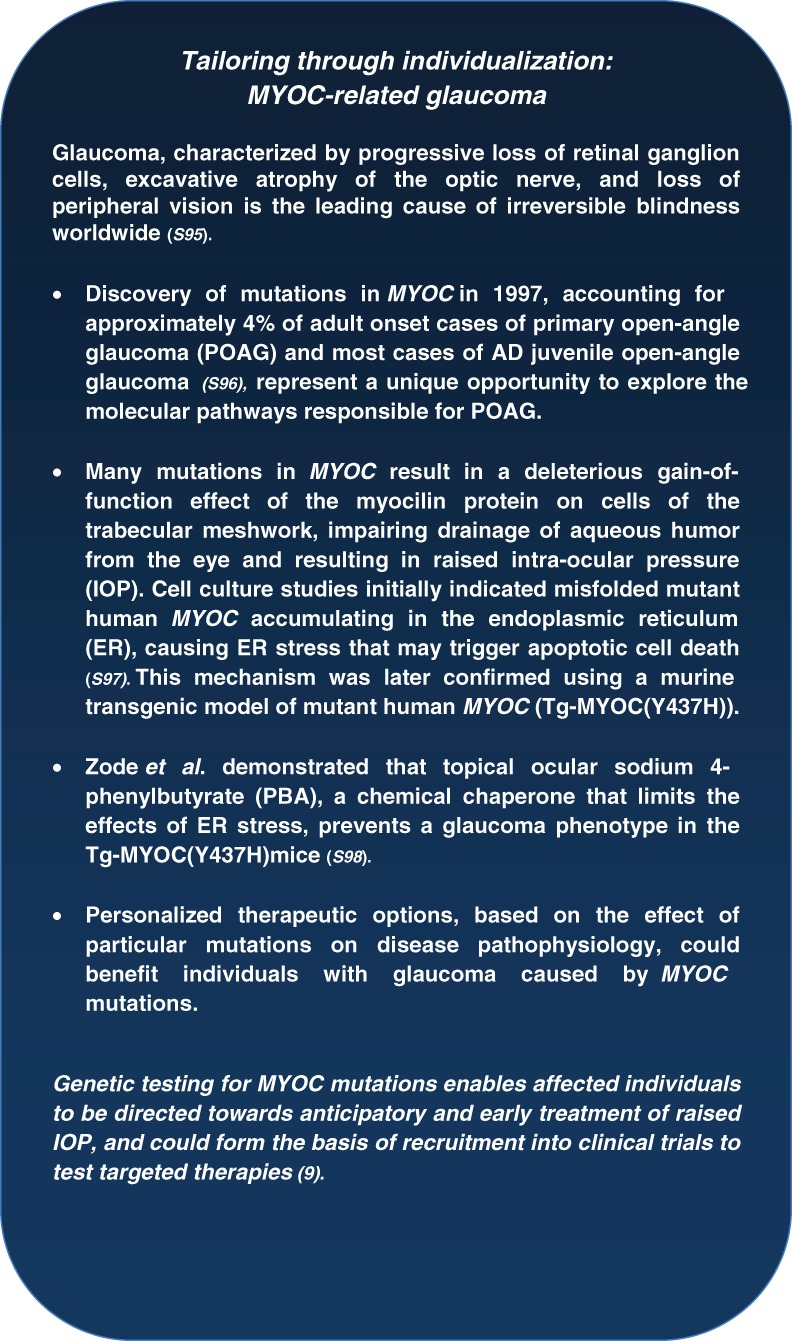
Tailoring through individualization: the case of glaucoma caused by *MYOC* mutations.

Mutations in six genes (*MYOC*, *PITX2*, *FOXC1*, *PAX6*, *CYP1B1*, and *LTBP2*) have been shown to cause overlapping phenotypes associated with congenital or juvenile glaucoma (S27), accounting for around 20% of cases of glaucoma with onset before the age of 40. The impact on clinical care and genetic counseling can be significant for those with a known genetic mutation because appropriate surveillance and timely treatment may prevent or limit sight loss 9 (Fig. 5). Indeed a study of 72 members of a family with *MYOC*-related glaucoma revealed that 96% of the family members benefited from genetic counseling and wished to know their myocilin status (9, S28). Detecting mutations helps to determine mode of inheritance; for example, mutations in *CYP1B1* and *LTBP2* cause recessive traits, whereas glaucoma caused by other known genes may be inherited in a dominant fashion. However incomplete penetrance and variable expressivity complicates recognition of the inheritance pattern (S27).

### Complex ophthalmic disease and personalized medicine: the case of age-related macular degeneration (AMD)

Complex disorders such as AMD and adult-onset primary open angle glaucoma (POAG) are less amenable to DNA-based diagnostic and predictive testing. While a number of high-risk AMD genotypes are documented (8, S29–S36) (Table2) evidence that genotyping for these variations can be clinically predictive remains limited. Currently standard clinical diagnostic methodologies (such as biomicroscopy, ophthalmoscopy, tomography and perimetry) are more accurate for assessing a person's risk of vision loss 10. Without proof that genotype-specific therapies are beneficial, it is probable that genetic testing for complex diseases will not become integrated into routine clinical practice at this stage  8,10.

**Table 2:** Age-related macular degeneration (AMD): delineating the genetic basis of complex disease. Genetic variants in AMD account for its high heritability (approximately 70% of total risk). Fifty percentage of the genetic risk is believed to be attributable to common variants – those highlighted in *CFH* (Y402H) and *ARMS2*/*HTRA1* are major contributors to AMD risk and pathogenesis. Identification of common variants has been performed using linkage analysis and large case–control studies (panels 1 and 2). The role of highly penetrant rare variants is more recent (lower panel). Highlighted is the association of AMD with *TIMP3*, a gene that also causes Sorsby fundus dystrophy

AMD: delineating the genetic basis of complex disease	
*Linkage analysis*/*candidate genes search*	*Case–control studies* (*GWAS*):
• 1q31: *CFH*	*Complement pathway genes*
• 10q26: *ARMS2*/*HTRA1*	• *C2*, *C3*, *C7*, *CFH*, *CFHR1*, *CFHR2*, *CFHR3*. *CFHR4*, *CFHR5*, *CFB*, *CFP*, *CFI*, *SERPING1*
• 4q25: *CFI*	• *ECM-related genes*
• 5p: *C7*	• *COL10A1*, *ELN*, *HMCN1*, *ARMS2*, *TIMP3*, *ROBO1*, *FBLN5*, *LOXL1*,*COL8A1*, *COL15A1*
• 6p: *C2*, *VEGF-A*	• *ADAMTS9*
• 6q21: *FRK*, *COL10A*	Lipid pathway genes
• 8p23: *LPL*	• *LIPC*, *APOE*, *LRP5*, *LRP6*, *VDLR*, *ABCA1*
• 9q: *ABCA1*, *COL15A1*, *TGFB1R*	*Other*
• 19p13: *C3*	• *TGFB1R*, *IGF1R*, *MYRIP*, *CACNG3*
• 22q: *TIMP-3*	
*Role of highly penetrant rare variants* (*NGS*)	
• *CFI* p.Gly119Arg, *C3* p.Lys155Gln, *CFH* p.Arg1210Cys, *C9* p.Pro167Ser	

AMD, age-related macular degeneration; *ARMS2*, the age-related maculopathy susceptibility 2; *CFH*, complement factor H; *HTRA1,* high-temperature requirement factor; NGS, next generation sequencing; GWAS, genome-wide association studies; *VEGF-A*, vascular endothelial growth factor.

AMD is the commonest cause of blindness in the Western world and is classified in two major forms, neovascular, or ‘wet’ AMD, and ‘dry’ or non-neovascular AMD (S37). The end-stage event in ‘wet’ AMD is choroidal neovascularization (CNV), an outgrowth of blood vessels from the choroid into the subretinal and intraretinal spaces leading to rapid loss of vision. In ‘dry’ AMD, geographic atrophy of the macula results from progressive retinal pigment epithelium (RPE) atrophy (S37). Neovascular AMD is responsible for 80% of severe vision loss and current treatments rely on pharmacological inhibition of vascular endothelial growth factor (VEGF)-A activity in those with overt disease (S38–S39).

Identification of genetic risk factors involved in AMD has been at the vanguard of genetic discovery for complex disease, providing important insights into disease pathogenesis. Family-based linkage studies, genome-wide association studies (GWAS) studies and candidate gene approaches identified major common variants conferring AMD risk on chromosomes 1q31 and 10q26 within, respectively, complement factor H (*CFH*) (Y402H) and the age-related maculopathy susceptibility 2 (*ARMS2*) and adjacent high-temperature requirement factor (*HTRA1*/*PRSS11*) genes (8, S30–S33). CFH is the main regulator of the alternative complement pathway and multiple independent genetic studies have now showed that dysfunction of the complement system is a key factor in AMD development 11. AMD is associated with risk variants in a number of other complement-related proteins including CFH-related proteins 1 and 3, factor B/C2, C3 and complement factor I (11, S40). Involvement of the complement system has been confirmed in immunohistochemical and proteomic studies on human donor eyes, and studies of blood complement levels in patients 11. The two main common short nucleotide polymorphism (SNP) associations on chromosomes 1q31 and 10q26 provide the greatest genetic contribution to AMD development risk (S33), but there remains no delineation either of genetic variants that predict disease progression, nor distinguish clearly between genetic variants causing neovascular or non-neovascular subphenotypes, respectively (S41–S43). The identification of high-risk subjects for targeting of lifestyle and dietary changes is attractive, but the challenges of using genetics for AMD risk prediction remain considerable, and such testing cannot yet be justified in the clinical arena.

### Tailoring through individualization

Disease stratification and provision of molecular diagnoses to our patients will impact positively upon many aspects of clinical management, enabling enhanced surveillance and development of early and anticipatory treatments in selected conditions, and, through genetic counseling, empower patient decision-making.

#### Genetic counseling

Genetic counseling is an important and integrative part of personalized medicine for genetic eye diseases. Ideally, patients should not undergo genetic testing without accessing counseling 10. Using non-directive principles, genetic counseling provides information to patients in a clear and unbiased manner, assisting them to make informed decisions. It precedes many personalized interventions such as carrier and predictive testing, recruitment into disease or mutation-specific databases, and forms an integral part of personalized reproductive medicine 12.

The impact of genetic testing on genetic counseling is significant, providing specificity to a diagnosis and clarifying inheritance patterns (12, S44–S45). Knowledge of the inheritance pattern is particularly important for patients with small families, and in our practice the majority of newly diagnosed IED patients have no known family history. Counseling is a fundamental aspect of pre-symptomatic testing, advantageous where preventative therapies exist, (Fig. 5) but also associated with more subtle potential benefits and risks when used to inform future decision-making. Where no effective therapies exist, caution is urged in the field of predictive testing for minors (10, S46–S47). Importantly, counseling explores an individual's motivation for testing 12.

#### Individualization of therapeutics

##### Conventional treatments

Molecular diagnosis enables optimization of conventional therapies for monogenic disorders. The number of conditions for which genetic profiling may improve traditional medical and surgical management are slowly increasing, and not only for ocular-related phenotypes. For example, delineation of the role of transforming growth factor-β (TFGβ) signaling in the genesis of aortic dissection in Marfan syndrome created unexpected avenues for treatment, leading to the use of losartan as a preventative agent (S48). Losartan is a selective angiotensin 1-receptor blocker, uniquely inhibiting TGFβ-mediated activation of extracellular signal-regulated kinase (ERK) while allowing continued signaling through the angiotensin 2 receptor, required for full protection against aneurysm progression in the mouse model (S48–S49).

Using conventional medicines to treat genetically heterogeneous diseases underlines the importance of a personalized approach. Advantages of molecular diagnosis in cases of CC associated with treatable systemic complications illustrate this point. Traditionally, diagnosis has relied on clinical suspicion (S50), diagnostic prediction in the post-genomic era enables individualized care pathways and early commencement of preventative treatments. Examples include the biochemical disorders cerebrotendinous xanthomatosis (CTX) and galactokinase deficiency, both associated with early-onset cataract. Patients who carry loss of function mutations in the *CYP27A1* gene develop CTX, a condition of lipid metabolism associated with juvenile cataracts, progressive neurological decline and cardiovascular disease usually diagnosed in mid adult life. Critically, early diagnosis is optimal because progression of cardiac and neurological phenotypes can be arrested by administration of chenodoxycholic acid in combination with statins (S50). Recessive mutations in *GALK1* cause galaktokinase deficiency with CC in around 75% of cases. Avoidance of galactose intake by strict dietary management may reduce and sometimes even reverse lenticular opacities (S51). Molecular diagnostics will increasingly achieve anticipatory diagnosis and alter the management of both presenting children and affected family members (S52).

##### Personalized therapeutics

The development of personalized therapeutics based on specific genetic predisposition is also envisaged. Treatments based on an individual (or tumor) genotype have been pioneered in oncology, using single genes or biomarkers to indicate tumors that will respond to specific chemotherapies (S53). Trastuzumab (Herceptin), a humanized monoclonal antibody that binds to the extracellular domain of human epidermal growth factor receptor 2 (HER2) is used for the treatment of breast cancer subtypes overexpressing HER2 and has revolutionized outcomes of patients with HER2-positive breast cancer. Several new HER2-targeted therapies and HER2/HER3 antibodies have now been developed for metastatic disease (S54).

Similar approaches have been proposed for complex ophthalmic disorders and insights gained from AMD genetics are providing the rationale for development of complement inhibitors for the treatment of AMD 11. Furthermore, there is interest in utilizing pharmacogenetics to predict treatment response in neovascular AMD. Intraocular injections of agents inhibiting VEGF are now the mainstay of treatment (S55–S56) and blindness rates have fallen since the mid-2000s (S57). However, a broad range of treatment response rates has been observed, possibly related to genetic factors (13, S58–S62). Chen et al., 2012 carried out a meta-analysis of small AMD pharmacogenetic studies and noted that *CFH* rs1061170 might be associated with treatment response of neovascular AMD to anti-VEGF agents (S62). Another recent study by Abedi et al. suggested a pharmacogenetic association between the *HTRA1* promoter SNP (rs11200638) and A69S at LOC387715/ARMS2 and anti-VEGF treatment 13. Response to the anti-VEGF agent ranibizumab has also been correlated to SNPs in VEGF genes and their receptors (VEGFR), particularly VEGFA and VEGFR2/KDR (S58). A study combining looking at at-risk AMD genotypes (SNPs in *CFH*, and *ARMS2*) with those SNPs identified in the angiogenesis pathway (SNPs in *VEGFA*, VEGF receptor *KDR*, *LRP5* and *FZD4*) showed that patients with four high-risk alleles in *CFH* and *ARMS2* were younger at age of onset of neovascular AMD and failed to show a visual improvement after ranibizumab treatment (S59). Although this approach has limited current benefit it may facilitate future treatment stratification 8.

##### Gene therapy

Currently, targeted therapies are emerging principally in the context of clinical trials for patients with inherited retinal dystrophies using gene-replacement strategies 7. The eye is said to be more amenable to gene therapy than many other organs, because of the structure and accessibility of the retina. This enables relatively non-invasive administration of the transducing agent and vigilant monitoring using simple clinical and imaging methods 7.

*RPE65*-associated Leber congenital amaurosis (LCA), a type of early-onset RP (Fig. 3), has been shown to be highly amenable to treatment with sub-retinal gene therapy in mice, dogs and humans (6 7, S11). In 2008, the first results of a phase I clinical trial using a gene replacement strategy for patients with LCA and *RPE65* mutations were published 6. These and further independent clinical trials showed proof-of-principle and some subjective benefits in navigational vision in some patients (6 14, S8, S10, S63). A 3-year follow up data on the five-patient Italian cohort involved in the LCA-*RPE65* gene therapy trial showed that improvement in visual acuity and retinal function was stable (S63). Dose-escalation trials ensued in younger patients confirming safety and highlighting visual improvements in children (S9–S10).

Choroideremia is an X-linked recessive disease caused by mutations in *REP1*, which encodes Rab escort protein 1(REP1). The condition is associated with night blindness, progressive peripheral to central chorioretinal degeneration and ultimately total blindness (S64). Rab proteins are involved in the control of intracellular trafficking and are modified by geranyl-geranyl moieties necessary for membrane association and target–protein recognition (S65–S66). Choroideremia is generally caused by *Rep1* null mutations or rare missense mutations affecting REP1 post-translational lipid modifications (S67–S68), suggesting that REP1-mediated Rab lipid modification (prenylation) is essential for RPE and photoreceptor function. Absence of protein or impaired post-translational modification is thought to affect opsin transport to photoreceptor outer segments, apical migration of RPE melanosomes and phagocytosis of photoreceptor outer segments by the RPE (S66–S68). Recent results from a phase 1/2 clinical gene-replacement trial are encouraging and suggest improved rod and cone function, with improvements in visual acuity, microperimetry and increases in retinal sensitivity in the treated eyes (S12).

Theoretical proof-of-principle of the benefits of targeted genetic therapies for inherited conditions is now established and offers promise for wider application to other retinal degenerations (S12) with ongoing pre-clinical studies for gene replacement strategies in other loss-of-function IRD such as *PDE6* AR RP (S69) and Bardet–Biedl syndromes caused by mutations in *BBS1* and *BBS4* (S70–S71).

However, the development of personalized therapies remains significantly limited by incomplete understanding of disease pathophysiology at the molecular level, highlighting the need for continued collaborative research efforts in this field.

## Challenges facing the adoption of personalized approaches to diagnosis

Increasing access to NGS and conventional sequencing technologies is providing unprecedented opportunities for improved clinical diagnosis and personalized interventions. At present genetic testing is particularly valuable when a treatment and counseling plan is dictated by the presence of a disease-causing mutation. This is currently the case for IRD, early onset glaucomas, and inherited optic neuropathies 9.

### Choice of diagnostic platform

Molecular diagnosis of genetic eye disease should be performed ideally with the most cost effective and simple modality available, and it is important to note that NGS will not be a stand-alone technology for advancing genomic medicine. Fluorescent *in situ* hybridization (FISH) and microarrays to detect copy number variations will remain important in the investigation of the dysmorphic or developmentally delayed infant with an ocular phenotype. For monogenic disorders, conventional sequencing will continue to play a role in the context of specific clinical diagnoses where there is accurate phenotypic data. For example in the case of the AR cone dystrophy associated with *KCNV2* mutations a characteristic phenotypic appearance is evident with patients showing supra-normal rod responses on electroretinogram (ERG) testing, directing molecular diagnosis (S72). Other retinal conditions with a characteristic phenotype include Doyne syndrome, where mutation screening of the affected gene can be performed seeking common or unique mutations.

Thus use of NGS platforms in the diagnosis of IRD, characterized by significant genetic and phenotypic heterogeneity discussed above, has been examined closely and pick-up rates and case selection is now well-understood (5 10, S18–S19). By contrast, the molecular diagnosis of developmental ocular disorders such as microphthalmia and anophthalmia is more complex. Only a minority of genetic causes is known and *de novo* mutations are common. The choice of whether to employ NGS on affected individuals, on trios (mother, father, child) and how to filter genomic data remains unclear for such disorders.

### Technological challenges of NGS

The increasing pace of NGS technologies presents enormous challenges in terms of data processing, storage and management, hindering translation from sequence data into clinical practice 3. Sequencing quality control and errors remain problematic, with uneven sequence coverage and quality across reads leading to patchy exome coverage. Platform-specific error profiles are evident, with many current platforms failing to detect in-frame deletions and deep intronic variants (S73). In addition, the amount of data generated using NGS demands a sophisticated computing infrastructure with skilled IT and bioinformatics staff to maintain and run NGS analysis tools. In addition, the cost of managing, storing and analyzing NGS data is currently a deterrent for adoption of NGS in many clinical settings (3, S3). These technological issues need to be addressed for the routine application of NGS (S73).

### NGS data interpretation

Our ability to understand the effects of identified variants on disease causation poses the greatest challenge to integration of NGS into clinical practice (15, S3). So far, interpretation of disease-causing variants has been carried out on a case-by-case basis, often in a research setting. The analysis is often based on software programs with information-based models that aim to predict pathogenicity of sequence changes. Such predictive software packages for functional analysis of variants have now been integrated into many NGS sequencing algorithms (S3). However, concerns remain over the accuracy of predictive software packages and most are limited to analyzing sequence changes within protein-coding exonic sequences (S74). Interpretation of missense changes, intronic and promoter variants remains suboptimal 15.

The gold standard proof of pathogenicity remains labor-intensive and expensive functional laboratory-based experiments. To bypass the need for such onerous validation, international sharing of variant data linked to standardized phenotypic descriptions is required to streamline data analysis for clinical use 15. This will require the creation of ‘pathogenicity-focused databases’ rather than simple repositories of human variation, that integrate patient clinical information and phenotype, genetic variants identified and pathogenicity data of the variants, along with the supporting evidence (PathoKB and Observ-OM database platforms) (15, S3).

### The need for testing guidelines

As knowledge of the number of genes mutated in eye disease increases, ophthalmologists who have training and experience in genetic eye disease and are supported by the necessary counseling infrastructure will increasingly order genetic tests and provide mechanism-specific treatments for their patients 10. There are now recommendations on the provision of genetic testing for patients with IED, aiming to increase its adoption and proposing that such testing be considered a routine element of clinical care 10.

### Need for accurate phenotyping

Increasingly, disease stratification will require accurate phenotyping alongside molecular diagnostics. As mentioned above, databases collecting clinical phenotypic information alongside gene specific variants and functional information are required to enable diagnostic context-specific data interpretation. Widespread interconnectivity between scientists and research-based data systems, diagnostics data systems and clinical patient-data systems will enable meaningful interaction between these three domains (S3). An example of this principle is the Café Variome (http:/www.cafevariome.org), deployed across sets of diagnostic laboratories with a common disease focus, as well as across groups of diagnostic laboratories from whole nations. Teams in this network can instantly find mutation records and rare disease patients recorded by other groups with summary data without releasing data or compromising patient privacy (S3).

### Consent and ethics

NGS carries with it a risk of collateral discovery of unrelated but clinically relevant findings, and the chance of making such a discovery is proportional to the amount of DNA assessed in each genetic test 10. NGS approaches are thus often undertaken in a clinical setting using a targeted approach to the analysis – only looking at genes for the specific disease referral (S3). The need for decisions on disclosure of unsolicited findings poses a challenge for the informed consent procedure (S75). Currently, consent for diagnostic exome sequencing often includes ‘opt-out’ options for unsolicited findings with the extent of return of results related to the mode of informed consent. In some cases advisory boards for unsolicited findings have been created. Guidelines are needed because both disclosure and non-disclosure of clinically relevant findings may undermine a patient's autonomy (S75).

### Drivers for adoption of personalized approaches

#### The need within medical research for translational outputs

There has been a rapid shift within biomedical research toward a culture of translation, increasing the focus on personalized approaches. Interest in rare monogenic diseases has run in parallel, recognizing opportunities to utilize potential novel therapies in the setting of specific conditions. Progress made in our understanding of the genetic basis of many IED represents a model of implementation and achievement in inherited disorders, with the creation of patient and mutation databases and global interdisciplinary projects already having a significant impact on the prospects of patients. In addition, however, more widespread adoption of personalized ophthalmology will require a shift in how evidence for utility is approached, because personalized medicine requires a ‘portfolio-based’ evidence base, encompassing retrospective analyses, prospective studies and comparative effectiveness, alongside randomized-controlled trials 1.

The development of targeted therapies will continue to challenge the ethical principles used in the selection of patients as candidates for novel medical therapeutics, with each new intervention assessed taking into account disease pathophysiology and mechanism of action of each tested intervention (S76). For example, many RP patients are still diagnosed when they have mid to late stage disease with peripheral vision loss as a consequence of photoreceptor degeneration. However, the success of clinical trials may depend on identifying patients as early as possible to maximize the efficacy of treatment (14, S69–S70) requiring changes in diagnostic processes as well as the introduction of asymptomatic individuals into clinical trials. The development of personalized therapeutics thus has broad implications within the wider medical culture, and not least faces the significant challenge of growing in a time of economic hardship for many public and privately funded health services.

#### Support from patients

Patients with IED provide support for the utility and validity of molecular diagnostic testing. While current availability of genetic testing for IED is variable within the publicly funded English National Health Service (NHS) for example (S26) – because of cost and perceived clinical utility (S77) – evidence for demand from service users is growing (S78–S79). Willis et al. showed in a questionnaire study of 200 patients with IRD that over 90% of participants were in favor of genetic testing for their disease, with support for both diagnostic (96.5%) and predictive testing (91.5%) being strong in these respondents (S44). A positive view of genetic testing in this population has been confirmed in other studies (S78–S79).

## Planning for future adoption of personalized approaches to ophthalmology

Proponents of personalized medicine suggest that reduced costs through better targeting of interventions will be a direct consequence. Certainly there is evidence for the cost-effectiveness of specific exemplars, for example *RB1* mutation testing in retinoblastoma-affected families (S80–S81) (Fig. 2); and use of genomic technologies in implementation of preventative strategies in specific areas, such as populations with high levels of consanguinity and a significant burden of autosomal recessive disease (S82). This example has led the World Health Organization (WHO) to recommend adoption of NGS to enhance genetic testing despite limited resources in some of these countries (S83).

However overall costs of delivering both disease stratification and treatment individualization cannot be overlooked. For example, while the cost of sequencing – on a per base level – has fallen rapidly in recent years 3 the increasing number of affected individuals and family members becoming eligible for testing 5 mean that it is probable that the overall cost of genomic testing within the clinical setting will rise rapidly. NGS costs must also include validation of identified variants via conventional sequencing, data storage and interpretation, with sequencing representing only a minority of the overall costs. When considering the cost of genetic testing, the whole pathway including family phenotyping, sequencing, data storage, analysis, interpretation and genetic counseling needs to be taken into account (10, S78).

Accelerated development of targeted therapies to run alongside improved diagnostic platforms is the ultimate hope of a personalized approach to healthcare. However the increasing costs of drug development for a tailored and therefore focused market will remain a huge hurdle reflected by the 75% increase in personalized medicine investment by industry over the past 5 years according to the Personalized Medicine Coalition (S87). The challenges of development – and critically, of equitable global application – of such therapies will be a huge future challenge. Although not a personalized therapy, the remarkable achievements of the FDA approved VEGF inhibitor ranibizumab in achieving disease control in neovascular AMD represents a triumph of molecular medicine, developed from a better understanding of the pathophysiology of AMD. It has provoked considerable escalation in the costs of treatment, with around 50,000 new patients per year becoming eligible for monthly injections in England alone at the cost of approximately 1500$ per injection (S84). Implementation difficulties of medical advances in Western healthcare systems are enormous. However application in developing systems is a further, often less well recognized problem. At this time, individualized therapies commercialized under the incentives introduced by the Orphan Drug Act, with substantial benefits and reductions in research and development costs, are setting precedents in terms of substantial annual costs (S85), generating acceleration in the orphan drug market, and increases in rates of approval of orphan drugs with a global orphan drug market of 84.9$billion in 2009 (S85, S87). The ability of such processes to keep pace with the speed of discovery remains in doubt, as is the sustainability of current pricing. Acceptable norms to control costs of personalized therapies – such as DNA-guided therapies – will be required, however decreasing profitability may result in drug companies developing fewer products for these until recently neglected diseases (S85). Personalized medicine, highly impacted by NGS with its multiple and diverse applications is profoundly influencing the drug development industry (S86). Overcoming these challenges will require input and consensus from governments, patients, charitable funders, scientists, industry and regulatory authorities and new business models for the pharmaceutical industry (S87).

Furthermore, service planning and delivery will have a deep impact on how patients and the medical profession respond to the development of personalized ophthalmology. The delivery of complex personalized care will require increased levels of clinical integration and communication. Ophthalmologists will need to be prepared to view genetic testing as an integral component of patient care, and be prepared to communicate effectively with laboratories and colleagues in medical genetics 10. The physical environment in which patients are seen will also increasingly play a part in making personalized ophthalmology a reality; integrated services that enable ophthalmologists to work in close liaison with counselors, medical geneticists and clinical diagnostic laboratories with access to new research findings will be required in many cases. Such a level of integration will allow research genetic tests to be offered with a focus on the transition from research to clinical testing. Access to investigative and management technologies including electrophysiology testing, ocular imaging, low vision services and patient support groups with information about emerging therapies will also need to be streamlined. Evidence for cost-effectiveness of integrated services is not widely available, but published evidence on the Ocular Genetics Program at the Toronto University Hospital shows that optimization of medical care for patients with ocular genetic disease with a cohesive structure, in one physical location, covering many aspects of patient's needs is possible, and patient satisfaction rates are extremely high (S88). Research for the advancement of personalized ophthalmology is also enhanced by integrated services as translational research depends on access to patients.

In conclusion, the prospects of personalized ophthalmology look promising, illustrated by the enhanced experiences of some patients with IED. Drawing on advances in new technologies to improve molecular diagnostics, basic and translational research to develop novel therapies, and fresh approaches to consent and data-sharing, personalized ophthalmology has the potential to revolutionize our specialty. Challenges developing individualized therapies in complex diseases such as AMD and glaucoma are significant, but AMD remains one of the finest examples of molecular dissection of a complex disease, positioning ophthalmology at the forefront of personalized therapies for complex diseases as they emerge. From a commercial point of view, AMD offers the promise of both precise and highly actionable personalized medicine, with the prospect of a sizeable market even for targeted therapies. While some ophthalmologists might question the immediate clinical relevance of molecular diagnostics, staying abreast of new technologies and their developing applications, and incorporating such testing into diagnostic, prognostic, and therapeutic algorithms of patient care when appropriate, will enable timely acquisition of sensitive and specific actionable interventions which improve patient care and outcome. Overcoming the challenges of developing personalized ophthalmology will require both global collaborative efforts integrating academic institutions, patient and clinical networks, regulatory authorities and commercial companies including the pharmaceutical industry. Finally, ophthalmologists themselves will consider the application of genomics and personalized therapies for their patients on a case-by-case basis, assessing clinically utility and cost-effectiveness, and will play a role in overseeing and promoting implementation of beneficial and equitable personalized healthcare advances for patients.

## Financial support

The authors would also like to acknowledge the support of the Manchester Academic Health Science Centre and the Manchester National Institute for Health Research Biomedical Research Centre. L. F. P. is a National Institute of Health (NIHR) research fellow.

## Supporting Information

The following Supporting information is available for this article:

Appendix S1. Supplemental references.
