# Supplementary material for: Personalized ophthalmology
Source: Clin Genet. 2014 Feb 9;86(1):1–11. doi: 10.1111/cge.12389 (PMC4232096; doi:10.1111/cge.12389)
Supplement: Supplementary file 1 — Appendix S1. Supplemental references. [file cge0086-0001-SD1.doc]

**Supplemental References**

S1. Berg M. Turning a practice into a science: reconceptualizing postwar medical practice. Soc Stud Sci 1995: 25: 437-476.

S2. Timmermans S and Berg M. The gold standard: the challenge of evidence-based medicine and standardization in health care. Philadelphia. Temple University Press, 2003.

S3. Ellard S, Patrinos GP, Oetting WS. Clinical applications of next-generation sequencing: the 2013 human genome variation society scientific meeting. Hum Mutat 2013: 34: 1583-1587.

S4. Williams RS, Willard HF, Snyderman R. Personalized health planning. Science 2003: 300: 549.

S5. Snyderman R. Personalized health care in 2013: A status report on the impact of genomics. N C Med J 2013: 74: 478-484.

S6. Bell CJ, Dinwiddie DL, Miller NA et al. Carrier testing for severe childhood recessive diseases by next-generation sequencing. Sci Transl Med 2011: 3: 65ra4.

S7. Maguire AM, Simonelli F, Pierce EA et al. Safety and efficacy of gene transfer for Leber’s congenital amaurosis. N Engl J Med 2008: 358: 2240-2248.

S8. [Cideciyan AV](http://www.ncbi.nlm.nih.gov/pubmed?term=Cideciyan AV%5BAuthor%5D&cauthor=true&cauthor_uid=19583479), [Hauswirth WW](http://www.ncbi.nlm.nih.gov/pubmed?term=Hauswirth WW%5BAuthor%5D&cauthor=true&cauthor_uid=19583479), [Aleman TS](http://www.ncbi.nlm.nih.gov/pubmed?term=Aleman TS%5BAuthor%5D&cauthor=true&cauthor_uid=19583479) et al. Human RPE65 gene therapy for Leber congenital amaurosis: persistence of early visual improvements and safety at 1 year. Hum Gene Ther 2009: 20 (9): 999-1004.

S9. [Maguire AM](http://www.ncbi.nlm.nih.gov/pubmed?term=Maguire AM%5BAuthor%5D&cauthor=true&cauthor_uid=19854499), [High KA](http://www.ncbi.nlm.nih.gov/pubmed?term=High KA%5BAuthor%5D&cauthor=true&cauthor_uid=19854499), [Auricchio A](http://www.ncbi.nlm.nih.gov/pubmed?term=Auricchio A%5BAuthor%5D&cauthor=true&cauthor_uid=19854499) et al. Age-dependent effects of RPE65 gene therapy for Leber's congenital amaurosis: a phase 1 dose-escalation trial. Lancet 2009: 374 (9701): 1597-605.

S10. [Jacobson SG](http://www.ncbi.nlm.nih.gov/pubmed?term=Jacobson SG%5BAuthor%5D&cauthor=true&cauthor_uid=21911650), [Cideciyan AV](http://www.ncbi.nlm.nih.gov/pubmed?term=Cideciyan AV%5BAuthor%5D&cauthor=true&cauthor_uid=21911650), [Ratnakaram R](http://www.ncbi.nlm.nih.gov/pubmed?term=Ratnakaram R%5BAuthor%5D&cauthor=true&cauthor_uid=21911650) et al. Gene therapy for Leber congenital amaurosis caused by RPE65 mutations: safety and efficacy in 15 children and adults followed up to 3 years. Arch Ophthalmol 2012: 130 (1): 9-24.

S11. [Beltran WA](http://www.ncbi.nlm.nih.gov/pubmed?term=Beltran WA%5BAuthor%5D&cauthor=true&cauthor_uid=22308428), [Cideciyan AV](http://www.ncbi.nlm.nih.gov/pubmed?term=Cideciyan AV%5BAuthor%5D&cauthor=true&cauthor_uid=22308428), [Lewin AS](http://www.ncbi.nlm.nih.gov/pubmed?term=Lewin AS%5BAuthor%5D&cauthor=true&cauthor_uid=22308428) et al. Gene therapy rescues photoreceptor blindness in dogs and paves the way for treating human X-linked retinitis pigmentosa. Proc Natl Acad Sci U S A 2012: 109 (6): 2132-7.

S12. [Maclaren RE](http://www.ncbi.nlm.nih.gov/pubmed?term=Maclaren RE%5BAuthor%5D&cauthor=true&cauthor_uid=24439297), [Groppe M](http://www.ncbi.nlm.nih.gov/pubmed?term=Groppe M%5BAuthor%5D&cauthor=true&cauthor_uid=24439297), [Barnard AR](http://www.ncbi.nlm.nih.gov/pubmed?term=Barnard AR%5BAuthor%5D&cauthor=true&cauthor_uid=24439297) et al. Retinal gene therapy in patients with choroideremia: initial findings from a phase 1/2 clinical trial. Lancet 2014: Jan 15 [Epub ahead of print].

S13. [Maclachlan TK](http://www.ncbi.nlm.nih.gov/pubmed?term=Maclachlan TK%5BAuthor%5D&cauthor=true&cauthor_uid=21119620), [Lukason M](http://www.ncbi.nlm.nih.gov/pubmed?term=Lukason M%5BAuthor%5D&cauthor=true&cauthor_uid=21119620), [Collins M](http://www.ncbi.nlm.nih.gov/pubmed?term=Collins M%5BAuthor%5D&cauthor=true&cauthor_uid=21119620) et al. Preclinical safety evaluation of AAV2-sFLT01- a gene therapy for age-related macular degeneration. Mol Ther 2011: 19(2): 326-34.

S14. Vincent A, Robson AG, Holder GE. Pathognomonic (diagnostic) ERGs. A review and update. Retina 2013: 33: 5-12.

S15. Wakabayashi T, Sawa M, Gomi F, Tsujikawa M. Correlation of fundus autofluorescence with photoreceptor morphology and functional changes in eyes with retinitis pigmentosa. Acta Ophthalmol 2010: 88: e177-e183.

S16. Mackay DS, Borman AD, Sui R et al. Screening of a large cohort of leber congenital amaurosis and retinitis pigmentosa patients identifies novel LCA5 mutations and new genotype-phenotype correlations. Hum Mutat 2013: 34: 1537-1546.

S17. Abu-Safieh L, Alrashed M, Anazi S et al. Autozygome-guided exome sequencing in retinal dystrophy patients reveals pathogenetic mutations and novel candidate disease genes. Genome Res 2013: 23: 236-247.

S18. Neveling K, Collin RW, Gilissen C et al. Next-generation genetic testing for retinitis pigmentosa. Hum Mutat 2012: 33: 963-972.

S19. Simpson DA, Clark GR, Alexander S, Silvestri G, Willoughby CE. Molecular diagnosis for heterogeneous genetic diseases with targeted high-throughput DNA sequencing applied to retinitis pigmentosa. J Med Genet 2011: 48: 145-151.

S20. Du WD, Chen G, Cao HM et al. A simple oligonucleotide biochip capable of rapidly detecting known mitochondrial DNA mutations in Chinese patients with Leber’s hereditary optic neuropathy (LHON). Dis Markers 2011: 30: 181-190.

S21. Gillespie RL, Hall G, Black GC. Genetic testing for inherited ocular disease: delivering on the promise at last? Clin Experiment Ophthalmol 2014: 42: 65-77.

S22. [Meyer BF](http://www.ncbi.nlm.nih.gov/pubmed?term=Meyer BF%5BAuthor%5D&cauthor=true&cauthor_uid=16096214). Strategies for the prevention of hereditary diseases in a highly consanguineous population**.** Ann Hum Biol 2005: 32 (2): 174-9.

S23. [Ahmed S](http://www.ncbi.nlm.nih.gov/pubmed?term=Ahmed S%5BAuthor%5D&cauthor=true&cauthor_uid=12374877), [Saleem M](http://www.ncbi.nlm.nih.gov/pubmed?term=Saleem M%5BAuthor%5D&cauthor=true&cauthor_uid=12374877), [Modell B](http://www.ncbi.nlm.nih.gov/pubmed?term=Modell B%5BAuthor%5D&cauthor=true&cauthor_uid=12374877), [Petrou M](http://www.ncbi.nlm.nih.gov/pubmed?term=Petrou M%5BAuthor%5D&cauthor=true&cauthor_uid=12374877). Screening extended families for genetic hemoglobin disorders in Pakistan. N Engl J Med 2002: 347 (15): 1162-8.

S24. [Hartong DT](http://www.ncbi.nlm.nih.gov/pubmed?term=Hartong DT%5BAuthor%5D&cauthor=true&cauthor_uid=17113430), [Berson EL](http://www.ncbi.nlm.nih.gov/pubmed?term=Berson EL%5BAuthor%5D&cauthor=true&cauthor_uid=17113430), [Dryja TP](http://www.ncbi.nlm.nih.gov/pubmed?term=Dryja TP%5BAuthor%5D&cauthor=true&cauthor_uid=17113430). Retinitis pigmentosa. Lancet 2006: 368 (9549): 1795-809.

S25. Koenekoop RK, Lopez I, den Hollander AI, Allikmets R, Cremers FP. Genetic testing for retinal dystrophies and dysfunctions: benefits, dilemmas and solutions. Clin Experiment Ophthalmol 2007: 35: 473-485.

S26. Moore T, Burton H. Genetic ophthalmology in focus: a needs assessment and review of specialist services for genetic eye disorders. 2008. April. <http://www.phgfoundation.org/reports/4983/>

S27. [Fan BJ](http://www.ncbi.nlm.nih.gov/pubmed?term=Fan BJ%5BAuthor%5D&cauthor=true&cauthor_uid=20811162), [Wiggs JL](http://www.ncbi.nlm.nih.gov/pubmed?term=Wiggs JL%5BAuthor%5D&cauthor=true&cauthor_uid=20811162). Glaucoma: genes, phenotypes, and new directions for therapy. J Clin Invest 2010: 120 (9): 3064-72.

S28. Healey DL, Craig JE, Wilkinson CH, Stone EM, Mackey DA. Attitudes to predictive DNA testing for myocilin glaucoma: experience with a large Australian family. J Glaucoma 2004: 13: 304-311.

S29. Klaver CC, Kliffen M, van Duijn CM et al. Genetic association of apolipoprotein E with age-related macular degeneration. Am J Hum Genet 1998: 63: 200-206.

S30. [Edwards AO](http://www.ncbi.nlm.nih.gov/pubmed?term=Edwards AO%5BAuthor%5D&cauthor=true&cauthor_uid=15761121), [Ritter R 3rd](http://www.ncbi.nlm.nih.gov/pubmed?term=Ritter R 3rd%5BAuthor%5D&cauthor=true&cauthor_uid=15761121), [Abel KJ](http://www.ncbi.nlm.nih.gov/pubmed?term=Abel KJ%5BAuthor%5D&cauthor=true&cauthor_uid=15761121), [Manning A](http://www.ncbi.nlm.nih.gov/pubmed?term=Manning A%5BAuthor%5D&cauthor=true&cauthor_uid=15761121), [Panhuysen C](http://www.ncbi.nlm.nih.gov/pubmed?term=Panhuysen C%5BAuthor%5D&cauthor=true&cauthor_uid=15761121), [Farrer LA](http://www.ncbi.nlm.nih.gov/pubmed?term=Farrer LA%5BAuthor%5D&cauthor=true&cauthor_uid=15761121). Complement factor H polymorphism and age-related macular degeneration. Science 2005: 308 (5720): 421-4.

S31. Klein RJ, Zeiss C, Chew EY et al. Complement factor H polymorphism in age-related macular degeneration. Science 2005: 308: 385-389.

S32. Rivera A, Fisher SA, Fritsche LG et al. Hypothetical LOC387715 is a second major susceptibility gene for age-related macular degeneration, contributing independently of complement factor H to disease risk. Hum Mol Genet 2005: 14: 3227-3236.

S33. Maller J, George S, Purcell S et al. Common variation in three genes, including a noncoding variant in CFH, strongly influences risk of age-related macular degeneration. Nat Genet 2006: 38: 1055-1059.

S34. Chen W, Stambolian D, Edwards AO et al. Genetic variants near TIMP3 and high-density lipoprotein-associated loci influence susceptibility to age-related macular degeneration. Proc Natl Acad Sci U S A 2010: 107: 7401-7406.

S35. Yu Y, Bhangale TR, Fagerness J et al. Common variants near FRK/COL10A1 and VEGFA are associated with advanced age-related macular degeneration. Hum Mol Genet 2011: 20: 3699-3709.

S36. Jun G, Nicolaou M, Morrison MA et al. Influence of ROBO1 and RORA on risk of age-related macular degeneration reveals genetically distinct phenotypes in disease pathophysiology. PLoS One 2011: 6: e25775.

S37. Eye Diseases Prevalence Research Group. Prevalence of age-related macular degeneration in the United States. Arch Ophthalmol 2004: 122: 564-72.

S38. [Aiello LP](http://www.ncbi.nlm.nih.gov/pubmed?term=Aiello LP%5BAuthor%5D&cauthor=true&cauthor_uid=7479819), [Pierce EA](http://www.ncbi.nlm.nih.gov/pubmed?term=Pierce EA%5BAuthor%5D&cauthor=true&cauthor_uid=7479819), [Foley ED](http://www.ncbi.nlm.nih.gov/pubmed?term=Foley ED%5BAuthor%5D&cauthor=true&cauthor_uid=7479819) et al. Suppression of retinal neovascularization in vivo by inhibition of vascular endothelial growth factor (VEGF) using soluble VEGF-receptor chimeric proteins. Proc Natl Acad Sci USA 1995: 92 (23): 10457-61.

S39. [Brown DM](http://www.ncbi.nlm.nih.gov/pubmed?term=Brown DM%5BAuthor%5D&cauthor=true&cauthor_uid=17893015), [Regillo CD](http://www.ncbi.nlm.nih.gov/pubmed?term=Regillo CD%5BAuthor%5D&cauthor=true&cauthor_uid=17893015). Anti-VEGF agents in the treatment of neovascular age-related macular degeneration: applying clinical trial results to the treatment of everyday patients. Am J Ophthalmol 2007: 144 (4): 627-37.

S40. [Seddon JM](http://www.ncbi.nlm.nih.gov/pubmed?term=Seddon JM%5BAuthor%5D&cauthor=true&cauthor_uid=24036952), [Yu Y](http://www.ncbi.nlm.nih.gov/pubmed?term=Yu Y%5BAuthor%5D&cauthor=true&cauthor_uid=24036952), [Miller EC](http://www.ncbi.nlm.nih.gov/pubmed?term=Miller EC%5BAuthor%5D&cauthor=true&cauthor_uid=24036952) et al. Rare variants in CFI, C3 and C9 are associated with high risk of advanced age-related macular degeneration. Nat Genet 2013: 45 (11): 1366-70.

S41. [Chakravarthy U](http://www.ncbi.nlm.nih.gov/pubmed?term=Chakravarthy U%5BAuthor%5D&cauthor=true&cauthor_uid=23098369), [McKay GJ](http://www.ncbi.nlm.nih.gov/pubmed?term=McKay GJ%5BAuthor%5D&cauthor=true&cauthor_uid=23098369), [de Jong PT](http://www.ncbi.nlm.nih.gov/pubmed?term=de Jong PT%5BAuthor%5D&cauthor=true&cauthor_uid=23098369) et al. ARMS2 increases the risk of early and late age-related macular degeneration in the European Eye Study. Ophthalmology 2013: 120 (2): 342-8.

S42. Hogg RE, McKay GJ, Hughes AE, Muldrew KA, Chakravarthy U. Genotype-phenotype associations in neovascular age-related macular degeneration. Retina 2012: 32: 1950-1958.

S43. Seddon JM, Francis PJ, George S, Schultz DW, Rosner B, Klein ML. Association of CFH Y402H and LOC387715 A69S with progression of age-related macular degeneration. JAMA 2007: 297: 1793-1800.

S44. Willis TA, Potrata B, Ahmed M et al. Understanding of and attitudes to genetic testing for inherited retinal disease: a patient perspective. Br J Ophthalmol 2013: 97: 1148-1154.

S45. Pelletier V, Jambou M, Delphin N et al. Comprehensive survey of mutations in RP2 and RPGR in patients affected with distinct retinal dystrophies: genotype-phenotype correlations and impact on genetic counseling. Hum Mutat 2007: 28: 81-91.

S46. Arribas-Ayllon M, Sarangi S, Clarke A. Professional ambivalence: accounts of ethical practice in childhood genetic testing. J Genet Couns 2009: 18: 173-184.

S47. Mezer E, Wygnanski-Jaffe T. Ethical issues in ocular genetics. Curr Opin Ophthalmol 2009: 20: 382-386.

S48. Gillis E, Van Laer L, Loeys BL. Genetics of thoracic aortic aneurysm: at the crossroad of transforming growth factor-beta signaling and vascular smooth muscle cell contractility. Circ Res 2013: 113: 327-340.

S49. Habashi JP, Doyle JJ, Holm TM et al. Angiotensin II type 2 receptor signaling attenuates aortic aneurysm in mice through ERK antagonism. Science 2011: 332: 361-365.

S50. Mignarri A, Gallus GN, Dotti MT, Federico A. A suspicion index for early diagnosis and treatment of cerebrotendinous xanthomatosis. J Inherit Metab Dis 2014: Jan 18. [Epub ahead of print]

S51. Bosch AM. Classical galactosaemia revisited. J Inherit Metab Dis 2006: 29: 516-525.

S52. Gillespie RL, Hall G, Black GC. Genetic testing for inherited ocular disease: delivering on the promise at last? Clin Experiment Ophthalmol 2014: 42: 65-77.

S53. Elborn JS. The impact of personalised therapies on respiratory medicine. Eur Respir Rev 2013: 22: 72-74.

S54. Figueroa-Magalhaes MC, Jelovac D, Connolly RM, Wolff AC. Treatment of HER2-positive breast cancer. Breast 2013: S0960-9776(13)00300-7. [Epub ahead of print]

S55. Rosenfeld PJ, Brown DM, Heier JS et al. Ranibizumab for neovascular age-related macular degeneration. N Engl J Med 2006: 355: 1419-1431.

S56. Wong TY, Liew G, Mitchell P. Clinical update: new treatments for age-related macular degeneration. Lancet 2007: 370: 204-206.

S57. Cheung N, Wong TY. Changing trends of blindness: the initial harvest from translational public health and clinical research in ophthalmology. Am J Ophthalmol 2012: 153: 193-195.

S58. [Hermann MM](http://www.ncbi.nlm.nih.gov/pubmed?term=Hermann MM%5BAuthor%5D&cauthor=true&cauthor_uid=24365177), [van Asten F](http://www.ncbi.nlm.nih.gov/pubmed?term=van Asten F%5BAuthor%5D&cauthor=true&cauthor_uid=24365177), [Muether PS](http://www.ncbi.nlm.nih.gov/pubmed?term=Muether PS%5BAuthor%5D&cauthor=true&cauthor_uid=24365177) et al. Polymorphisms in Vascular Endothelial Growth Factor Receptor 2 Are Associated with Better Response Rates to Ranibizumab Treatment in Age-related Macular Degeneration. Ophthalmology 2013: S0161-6420 (13) 01056-7.

S59. [Smailhodzic D](http://www.ncbi.nlm.nih.gov/pubmed?term=Smailhodzic D%5BAuthor%5D&cauthor=true&cauthor_uid=22840423), [Muether PS](http://www.ncbi.nlm.nih.gov/pubmed?term=Muether PS%5BAuthor%5D&cauthor=true&cauthor_uid=22840423), [Chen J](http://www.ncbi.nlm.nih.gov/pubmed?term=Chen J%5BAuthor%5D&cauthor=true&cauthor_uid=22840423) et al. Cumulative effect of risk alleles in CFH, ARMS2, and VEGFA on the response to ranibizumab treatment in age-related macular degeneration. Ophthalmology 2012: 119 (11): 2304-11.

S60. Hagstrom SA, Ying GS, Pauer GJ et al. Pharmacogenetics for genes associated with age-related macular degeneration in the Comparison of AMD Treatments Trials (CATT). Ophthalmology 2013: 120: 593-599.

S61. Orlin A, Hadley D, Chang W et al. Association between high-risk disease loci and response to anti-vascular endothelial growth factor treatment for wet age-related macular degeneration. Retina 2012: 32: 4-9.

S62. Chen H, Yu KD, Xu GZ. Association between variant Y402H in age-related macular degeneration (AMD) susceptibility gene CFH and treatment response of AMD: a meta-analysis. PLoS One 2012: 7: e42464.

S63. [Testa F](http://www.ncbi.nlm.nih.gov/pubmed?term=Testa F%5BAuthor%5D&cauthor=true&cauthor_uid=23474247), [Maguire AM](http://www.ncbi.nlm.nih.gov/pubmed?term=Maguire AM%5BAuthor%5D&cauthor=true&cauthor_uid=23474247), [Rossi S](http://www.ncbi.nlm.nih.gov/pubmed?term=Rossi S%5BAuthor%5D&cauthor=true&cauthor_uid=23474247) et al. Three-year follow-up after unilateral subretinal delivery of adeno-associated virus in patients with Leber congenital Amaurosis type 2. Ophthalmology 2013: 120 (6): 1283-91.

S64. [Cremers FP](http://www.ncbi.nlm.nih.gov/pubmed?term=Cremers FP%5BAuthor%5D&cauthor=true&cauthor_uid=2215697), [van de Pol DJ](http://www.ncbi.nlm.nih.gov/pubmed?term=van de Pol DJ%5BAuthor%5D&cauthor=true&cauthor_uid=2215697), [van Kerkhoff LP](http://www.ncbi.nlm.nih.gov/pubmed?term=van Kerkhoff LP%5BAuthor%5D&cauthor=true&cauthor_uid=2215697), [Wieringa B](http://www.ncbi.nlm.nih.gov/pubmed?term=Wieringa B%5BAuthor%5D&cauthor=true&cauthor_uid=2215697), [Ropers HH](http://www.ncbi.nlm.nih.gov/pubmed?term=Ropers HH%5BAuthor%5D&cauthor=true&cauthor_uid=2215697). Cloning of a gene that is rearranged in patients with choroideraemia. Nature 1990: 347 (6294): 674-7.

S65. Jacobson SG, Cideciyan AV, Sumaroka A et al. Remodeling of the human retina in choroideremia: rab escort protein 1 (REP-1) mutations. Invest Ophthalmol Vis Sci 2006: 47: 4113-4120.

S66. [Alory C](http://www.ncbi.nlm.nih.gov/pubmed?term=Alory C%5BAuthor%5D&cauthor=true&cauthor_uid=11489211), [Balch WE](http://www.ncbi.nlm.nih.gov/pubmed?term=Balch WE%5BAuthor%5D&cauthor=true&cauthor_uid=11489211). Organization of the Rab-GDI/CHM superfamily: the functional basis for choroideremia disease. Traffic 2001: 2 (8): 532-43.

S67. [Sergeev YV](http://www.ncbi.nlm.nih.gov/pubmed?term=Sergeev YV%5BAuthor%5D&cauthor=true&cauthor_uid=19427510), [Smaoui N](http://www.ncbi.nlm.nih.gov/pubmed?term=Smaoui N%5BAuthor%5D&cauthor=true&cauthor_uid=19427510), [Sui R](http://www.ncbi.nlm.nih.gov/pubmed?term=Sui R%5BAuthor%5D&cauthor=true&cauthor_uid=19427510) et al. The functional effect of pathogenic mutations in Rab escort protein 1. Mutat Res 2009: 665(1-2): 44-50.

S68. Esposito G, De Falco F, Tinto N et al. Comprehensive mutation analysis (20 families) of the choroideremia gene reveals a missense variant that prevents the binding of REP1 with Rab geranylgeranyl transferase. Hum Mutat 2011: 32: 1460-1469.

S69. [Davis RJ](http://www.ncbi.nlm.nih.gov/pubmed?term=Davis RJ%5BAuthor%5D&cauthor=true&cauthor_uid=23946405), [Hsu CW](http://www.ncbi.nlm.nih.gov/pubmed?term=Hsu CW%5BAuthor%5D&cauthor=true&cauthor_uid=23946405), [Tsai YT](http://www.ncbi.nlm.nih.gov/pubmed?term=Tsai YT%5BAuthor%5D&cauthor=true&cauthor_uid=23946405) et al. Therapeutic margins in a novel preclinical model of retinitis pigmentosa. J Neurosci 2013: 33 (33): 13475-83.

S70. Seo S, Mullins RF, Dumitrescu AV et al. Subretinal gene therapy of mice with Bardet-Biedl syndrome type 1. Invest Ophthalmol Vis Sci 2013: 54: 6118-6132.

S71. Simons DL, Boye SL, Hauswirth WW, Wu SM. Gene therapy prevents photoreceptor death and preserves retinal function in a Bardet-Biedl syndrome mouse model. Proc Natl Acad Sci U S A 2011: 108: 6276-6281.

S72. Zelinger L, Wissinger B, Eli D, Kohl S, Sharon D, Banin E. Cone dystrophy with supernormal rod response: novel KCNV2 mutations in an underdiagnosed phenotype. Ophthalmology 2013: 120: 2338-2343.

S73. Taub MA, Corrada Bravo H, Irizarry RA. Overcoming bias and systematic errors in next generation sequencing data. Genome Med 2010: 2: 87.

S74. Joppa LN, McInerny G, Harper R et al. Computational science. Troubling trends in scientific software use. Science 2013: 340: 814-815.

S75. [Rigter T](http://www.ncbi.nlm.nih.gov/pubmed?term=Rigter T%5BAuthor%5D&cauthor=true&cauthor_uid=24117109), [van Aart C](http://www.ncbi.nlm.nih.gov/pubmed?term=van Aart C%5BAuthor%5D&cauthor=true&cauthor_uid=24117109), [Elting M](http://www.ncbi.nlm.nih.gov/pubmed?term=Elting M%5BAuthor%5D&cauthor=true&cauthor_uid=24117109), [Waisfisz Q](http://www.ncbi.nlm.nih.gov/pubmed?term=Waisfisz Q%5BAuthor%5D&cauthor=true&cauthor_uid=24117109), [Cornel M](http://www.ncbi.nlm.nih.gov/pubmed?term=Cornel M%5BAuthor%5D&cauthor=true&cauthor_uid=24117109), [Henneman L](http://www.ncbi.nlm.nih.gov/pubmed?term=Henneman L%5BAuthor%5D&cauthor=true&cauthor_uid=24117109). Informed consent for exome sequencing in diagnostics: exploring first experiences and views of professionals and patients. Clin Genet 2013: doi: 10.1111/cge.12299. [Epub ahead of print]

S76. [Lowenstein PR](http://www.ncbi.nlm.nih.gov/pubmed?term=Lowenstein PR%5BAuthor%5D&cauthor=true&cauthor_uid=18956015). A call for physiopathological ethics. Mol Ther. 2008: 16 (11): 1771-2.

S77. Eden M, Payne K, Combs RM, Hall G, McAllister M, Black GC. Valuing the benefits of genetic testing for retinitis pigmentosa: a pilot application of the contingent valuation method. Br J Ophthalmol 2013: 97: 1051-1056.

S78. Combs R, McAllister M, Payne K et al. Understanding the impact of genetic testing for inherited retinal dystrophy. Eur J Hum Genet 2013: 21: 1209-1213.

S79. Pradhan M, Hayes I, Vincent A. An audit of genetic testing in diagnosis of inherited retinal disorders: a prerequisite for gene-specific intervention. Clin Experiment Ophthalmol 2009: 37: 703-711.

S80. Richter S, Vandezande K, Chen N et al. Sensitive and efficient detection of RB1 gene mutations enhances care for families with retinoblastoma. Am J Hum Genet 2003: 72: 253-269.

S81. Noorani HZ, Khan HN, Gallie BL, Detsky AS. Cost comparison of molecular versus conventional screening of relatives at risk for retinoblastoma. Am J Hum Genet 1996: 59: 301-307.

S82. Kingsmore SF, Lantos JD, Dinwiddie DL et al. Next-generation community genetics for low- and middle-income countries. Genome Med 2012: 4: 25.

S83. WHO. Community genetics services: report of a WHO consultation on community genetics in low-and-middle-income countries, 2010. Available online: whqlibdoc.who.int/publications/2011/9789241501149.

S84. Chakravarthy U, Harding SP, Rogers CA et al. Alternative treatments to inhibit VEGF in age-related choroidal neovascularisation: 2-year findings of the IVAN randomised controlled trial. Lancet 2013: 382: 1258-1267.

S85. Barrett PM, Alagely A, Topol EJ. Cystic fibrosis in an era of genomically guided therapy. Hum Mol Genet 2012: 21: R66-R71.

S86. Woollard PM, Mehta NA, Vamathevan JJ, Van Horn S, Bonde BK, Dow DJ. The application of next-generation sequencing technologies to drug discovery and development. Drug Discov Today 2011: 16: 512-519.

S87. Mesko B, Zahuczky G, Nagy L. The triad of success in personalised medicine: pharmacogenomics, biotechnology and regulatory issues from a Central European perspective. N Biotechnol 2012: 29: 741-750.

S88. Morad Y, Sutherland J, DaSilva L et al. Ocular Genetics Program: multidisciplinary care of patients with ocular genetic eye disease. Can J Ophthalmol 2007: 42: 734-738.

**FIGURE references**

**Figure 2.**

S89. American Society of Clinical Oncology policy statement update: genetic testing for cancer susceptibility. J Clin Oncol 2003: 21(12): 2397-406.

S90. Lohmann D, Scheffer H, Gaille B. Best Practice Guidelines for Molecular Analysis of Retinoblastoma. European Molecular Genetics Quality Network. Available online 2002. [www.emqn.org/emqn/digitalAssets/0/239_RB.pdf](http://www.emqn.org/emqn/digitalAssets/0/239_RB.pdf)

**Figure 4:**

S91. Sparrow JR, Vollmer-Snarr HR, Zhou J et al. A2E-epoxides damage DNA in retinal pigment epithelial cells. Vitamin E and other antioxidants inhibit A2E-epoxide formation. The Journal of biological chemistry 2003: 278: 18207-13.

S92. Stone EM, Lotery AJ, Munier FL et al. A single EFEMP1 mutation associated with both Malattia Leventinese and Doyne honeycomb retinal dystrophy. Nat Genet 1999: 22: 199-202.

S93. Weber BH, Vogt G, Pruett RC, Stohr H, Felbor U. Mutations in the tissue inhibitor of metalloproteinases-3 (TIMP3) in patients with Sorsby's fundus dystrophy. Nat Genet 1994: 8: 352-356.

S94. Boon CJ, Klevering BJ, Leroy BP, Hoyng CB, Keunen JE, den Hollander AI. The spectrum of ocular phenotypes caused by mutations in the BEST1 gene. Prog Retin Eye Res 2009: 28: 187-205.

**Figure 5.**

S95. Kwon YH, Fingert JH, Kuehn MH et al. Primary open-angle glaucoma*.* N Engl J Med 2009: 360(11): 1113-24.

S96. Stone EM, Fingert JH, Alward WL et al. Identification of a gene that causes primary open angle glaucoma. Science 1997: 275: 668-670.

S97. Shepard AR, Jacobson N, Millar JC et al. Glaucoma-causing myocilin mutants require the Peroxisomal targeting signal-1 receptor (PTS1R) to elevate intraocular pressure. Hum Mol Genet 2007: 16: 609-617.

S98. [Zode GS](http://www.ncbi.nlm.nih.gov/pubmed?term=Zode GS%5BAuthor%5D&cauthor=true&cauthor_uid=22328638), [Bugge KE](http://www.ncbi.nlm.nih.gov/pubmed?term=Bugge KE%5BAuthor%5D&cauthor=true&cauthor_uid=22328638), [Mohan K](http://www.ncbi.nlm.nih.gov/pubmed?term=Mohan K%5BAuthor%5D&cauthor=true&cauthor_uid=22328638) et al. Topical ocular sodium 4-phenylbutyrate rescues glaucoma in a myocilin mouse model of primary open-angle glaucoma. Invest Ophthalmol Vis Sci 2012: 53 (3): 1557-65.
